# Downregulation of β-Adrenoceptors in Isoproterenol-Induced Cardiac Remodeling through HuR

**DOI:** 10.1371/journal.pone.0152005

**Published:** 2016-04-01

**Authors:** Qian Yin, Chengzhi Yang, Jimin Wu, Haiyan Lu, Xiaohui Zheng, Youyi Zhang, Zhizhen Lv, Xiaopu Zheng, Zijian Li

**Affiliations:** 1 Department of Cardiovascular Medicine, the First Affiliated Hospital of Xi'an Jiaotong University, Xi’an 71006, China; 2 Institute of Vascular Medicine, Peking University Third Hospital, Beijing Key Laboratory of Cardiovascular Receptors Research, Key Laboratory of Cardiovascular Molecular Biology and Regulatory Peptides, Ministry of Health and Key Laboratory of Molecular Cardiovascular Sciences, Ministry of Education, Beijing, 100191, China; 3 Key Laboratory of Resource Biology and Biotechnology in Western China, Ministry of Education, College of Life Sciences, Northwest University, Xi’an 710069, China; 4 Key laboratory of Chinese internal medicine of MOE and Beijing, Dongzhimen hospital, Beijing university of Chinese medicine, 5# Haiyuncang lane, Dongcheng district, Beijing100700, China; Temple University, UNITED STATES

## Abstract

β-adrenergic receptors (β-ARs) play an important role in cardiac remodeling, which is the key pathological process in various heart diseases and leads to heart failure. However, the regulation of β-AR expression in remodeling hearts is still unclear. This study aims to clarify the possible mechanisms underlying the regulation of β_1_- and β_2_-AR expression in cardiac remodeling. The rat model of cardiac remodeling was established by subcutaneous injection of isoproterenol(ISO) at the dose of 0.25 mg·kg^−1^·d^−1^ for 7days. We found that the expression of β_1_- and β_2_-ARs decreased in the remodeling heart. The mechanisms may include the inhibition of DNA transcription and the increase of mRNA degradation. cAMP-response element binding protein(CREB) is a well-known transcription factor of β-AR. However, the expression and activation of CREB was not changed in the remodeling heart. Further, human Antigen-R (HuR), a RNA binding protein, which binds to the 3'-untranslated region of the β-AR mRNA and promotes RNA degradation, was increased in the remodeling model. And in *vitro*, HuR deficiency reversed the reduction of β-AR mRNA induced by ISO. Therefore, the present findings indicate that HuR, but not CREB, is responsible for the reduction of β-AR expression in ISO induced cardiac remodeling.

## Introduction

Cardiac remodeling referrers mainly to cardiac myocyte hypertrophy and interstitial fibrosis, which results in the pathologic and functional alterations of heart and promotes the progression of heart failure[1], ventricular arrhythmia and sudden death[2]. The activated sympathoadrenal system, involving the sympathetic nervous system and the adrenal medulla, participates in the development of cardiac remodeling through the elevation of plasma catecholamines and the sustained activation of cardiac adrenergic receptors (ARs)[3]. The major subtype of adrenoceptors in heart is β-AR, which comprises roughly 90% of the total cardiac ARs[4]. Isoproterenol(ISO), a non-selective β-AR agonist, was widely used to establish cardiac remodeling model by inducing the sustained activation of β-AR[5, 6].

The change in β-AR expression is important to signaling alteration[7] in the process of cardiac remodeling. The myocardial β-AR density was increased in TGF-β_1_ induced hypertrophy [8]. In addition, an increase of β_2_-AR expression and a decrease of β_1_-AR was showed at the early stage of Doxorubicin-induced cardiomyopathy[9]. However, the change of β-AR expression in remodeling heart caused by sustained adrenergic activation and the molecular mechanism are not well understood.

Gene expression is regulated at both transcriptional and post-transcriptional levels. cAMP-response element binding protein(CREB), a 43-kD basic leucine zipper transcription factor, regulates the transcription of many genes through binding to the CRE, an 8-bp palindromic consensus element (TGACGTCA), in their promoters[10].CREB also regulates the transcription of β-AR[11, 12]. Further, the mRNA stability is important to the post-transcriptional regulation. Hu antigen R (HuR) recognizes reiterated AUUUA sequences[13] and has been implicated in the regulation of RNA stability of β_1_- and β_2_-AR through binding to their UTRs [14–18].

In the present study, we investigated the expression of β_1_-and β_2_-ARs in ISO-induced cardiac remodeling and the regulation mechanisms at transcriptional and post-transcriptional levels.

## Materials and Methods

### Animals and treatment

In the present experiments, 14 SD rats of 6–8 weeks old, weighing 280–320g, were randomly divided into two groups, ISO group and vehicle group. In ISO group, rats were treated with subcutaneous injection of isoproterenol(0.25 mg·kg^−1·^d^−1^, dissolved in saline, Sigma–Aldrich, St. Louis, MO, USA) once daily for seven consecutive days. In vehicle group, rats were infused with saline as the control. All animals care and experimental procedures were approved by the Institutional Animal Care and Use Committee of Peking University Health Science Center. Rats were housed in a temperature-and humidity-controlled room on a 12 h light/dark cycle and given free access to water and food. All studies involving animals are reported in accordance with the ARRIVE guidelines for reporting experiments involving animals[19, 20].

### Echocardiography

The transthoracic echocardiography was performed using a Visualsonicshigh-resolution Vevo 770 system (VisualSonics Inc., Toronto, Canada) equipped with a 30 MHz sectorial probe. Rats were anaesthetized with gas-mixture of 3% isoflurane(Baxter Healthcare Corp, New Providence, RI, USA).And rats were positioned on a heating platform with a small dose of isoflurane to stabilize physiological values especially heart rate.

The parasternal long-axis image was viewed with B-mode, and the parasternal short-axis(SAX) image at the level of papillary muscles was acquired when the scanhead was rotated clockwise 90°. Then, the M-mode image was obtained using the M-mode cursor to measure wall thickness in the end-diastole and systole of left ventricular. We took ejection fraction(EF%), fractional shortening(FS%), LV anterior wall thickness at diastole and systole (LVAWd and LVAWs, respectively) and LV posterior wall thickness at diastole and systole(LVPWd and LVPWs, respectively) as recommended by the American Society of Echocardiography. Measurements were made on three continuous cardiac cycles per loop and averaged for each data.

### Histological analysis

Heart tissues fixed with 10% formalin(PH 7.4) were embedded in paraffin, sectioned into 5-μm slices, and stained with wheat germ agglutinin(WGA).The myocytes cross-sectional areas were analyzed by Image-Pro Plus (Media Cybernetics,U.S.A) and at least 50 cardiomyocytes were measured in one mid-ventricular cross-section. The sections were stained with picrosirius red. The ratio of sirius red-stained area to total ventricular area, termed as the collagen volume fraction (CVF), was calculated by Image-Pro Plus. And we randomly selected 10 fields from each section.

### Immunohistochemistry

Heart tissues were fixed with 4% formaldeyde, embeded in paraffin, and made in 5-μm slices. Antigen retrieval were measured by heating sections at 92–93°C for 10minutes in natrium citricum buffer(PH = 6.0). The sections were blocked with 5% goat serum and incubated with the primary antibody of β_1_-AR and HuR(Santa Cruz Biotechnology, Dallas, Texas, USA)at 4°C overnight. Afterward, sections were incubated with the secondary antibody(Zhongshan Golden Bridge Biotechnology, Beijing, China) at 37°C for 1h, visualized with the DAB substrate system(Zhongshan Golden Bridge Biotechnology Laboratories) and counterstained with hematoxylin. The sections were then photographed with the Leica Q500 IW light microscope(Leica, Solms,Germany).

### Immunofluorescence

Heart tissues were fixed with 4% formaldeyde, embeded in paraffin, and made in 5-μm slices. After antigen retrieval, the sections were blocked with 5%BSA (Life Technologies, U.S.A) and incubated with the primary antibody ofβ_2_-AR(abcam,Cambridge,U.S.) at 4°C overnight. The second antibody, donkey anti-rabbit Alexa Fluor 568(Life Technologies, Grand Island, NY,USA),was incubated at room temperature for 1h and nuclei were counterstained with Hoechst 33258. Images were acquired using Zeiss LSM 780 confocal microscope (Zeiss, Oberkochen, Baden-Württemberg,German).

### Real time-PCR

Total RNA was isolated from the left ventricle using TRIzol reagent (Invitrogen, Carlsbad, CA, USA) and quantified by measuring the absorbance at 260nm. One microgram of total RNA was used for reverse transcription. And rat type I collagen, rat type III collagen, ANP, β_1_-AR, β_2_-AR and CREB were determined by real time PCR (Eppendorf Mastercycler ep realplex, Eppendorf, Hamburg,Germany). The reaction conditions involved denaturation at 95°C for15s, annealing at 60°C for 30s,and extension at 72°C for 30s. The PCR products were sequenced. The gene expression level was calculated by 2^-ΔCT^. Primers were shown in the Table 1.

**Table 1:** Primer sequence.

Target gene	Primer sequence(5’-3’)
Collagen-I (F)	ATCAGCCCAAACCCCAAGGAGA
Collagen-I (R)	CGCAGGAAGGTCAGCTGGATAG
Collagen-III (F)	TGATGGGATCCAATGAGGGAGA
Collagen-III (R)	GAGTCTCATGGCCTTGCGTGTTT
ANP(F)	CCTGGACTGGGGAAGTCAAC
ANP(R)	GTCAATCCTACCCCCGAAGC
β_1_-AR(F)	CTGCCCTTTCGCTACCAGAG
β_1_-AR(R)	ACTGGGGTCGTTGTAGCAG
β_2_-AR(F)	GAGACCCTGTGCGTGATTGC
β_2_-AR(R)	CCTGCTCCACCTGGCTGAGG
CREB(F)	TGCGACTGAGCAGGACATAG
CREB(R)	ATGACGAGGGACTGAGCAGA
GAPDH (F)	TCCCTCAAGATTGTCAGCAA
GAPDH (R)	AGATCCACAACGGATACATT
β-actin(F)	AGGGAAATCGTGCGTGACAT
β-actin(R)	AACCGCTCATTGCCGATAGT

### Western-blot

The expressions of β_1_-AR(Santa Cruz Biotechnology), β_2_-AR(Santa Cruz Biotechnology), phospho-CREB, CREB(Cell Signaling Technology, Danvers, Massachusetts, USA) and HuR(Santa Cruz Biotechnology) were examined by western-blot. All cell samples were lysed in lysis buffer. The protein concentration was assessed by BCA protein assay kit (Life Technologies). Proteins were subjected to electrophoresis with 10% SDS polyacrylamide gel and transferred to NC membranes. The membranes were analyzed with antibodies according to the supplier’s protocol, and immunolabelled bands were visualized by use of the Pierce ECL Western Blotting Substrate (Thermo Fisher Scientific, San Jose, CA, USA).

### Neonatal rat cardiac myocytes (NRCMs) culture

Primary neonatal rat cardiac myocytes (NRCMs) were harvested from ventricles of 1–3 day-old rats as previously described [21]. Ventricles were minced and digested with 0.01% collagenase II (Worthington, Columbia, NJ, U.S.A.). Cells were collected and plated for 2h at 37°C. Cardiomyocytes, the unattached cells, were removed in a new dish and cultured in Dulbecco’s modified Eagle’s medium (DMEM) (Gibco, USA) with 10% FBS(Hyclone Laboratories, Omaha, NE, U.S.A.), 100U/mL penicillin, 100μg/mL streptomycin at 37°C with 5% CO2.

### Assessment of HuR knockdown

The specific siRNA targeting rat HuR were synthesized with the sequence 5’-AAG AGG CAA UUA CCA GUU UCA -3’. The scrambled sequences were synthesized with the sequence 5’-UUC UCC GAA CGU GUC ACG UTT-3’.The day before transfection, cells were seeded in 6 well plate in 10%FBS/DMEM without antibiotics and grew overnight. At the day of transfection, ScreenFect^TM^A(Incella, Germany)-siRNA complexes were prepared in dilution buffer according to manufacturer’s protocol. Cells were transfected with either 100nMHuR siRNAs or scrambled siRNA complexes for 24h in opti-MEM I (life technologies, U.S.A). HuR expression was assessed by western blot.

### Statistics

Results were expressed as mean±SEM and all data were analyzed with Graph Pad Prism 5(GraphPad Software,La Jolla,CA,USA). t-test and two-way ANOVA were used to compare differences, P <0.05 was considered to be statistically significant between groups.

## Results

### Cardiac hypertrophy induced by ISO stimulation

**Fig 1:**
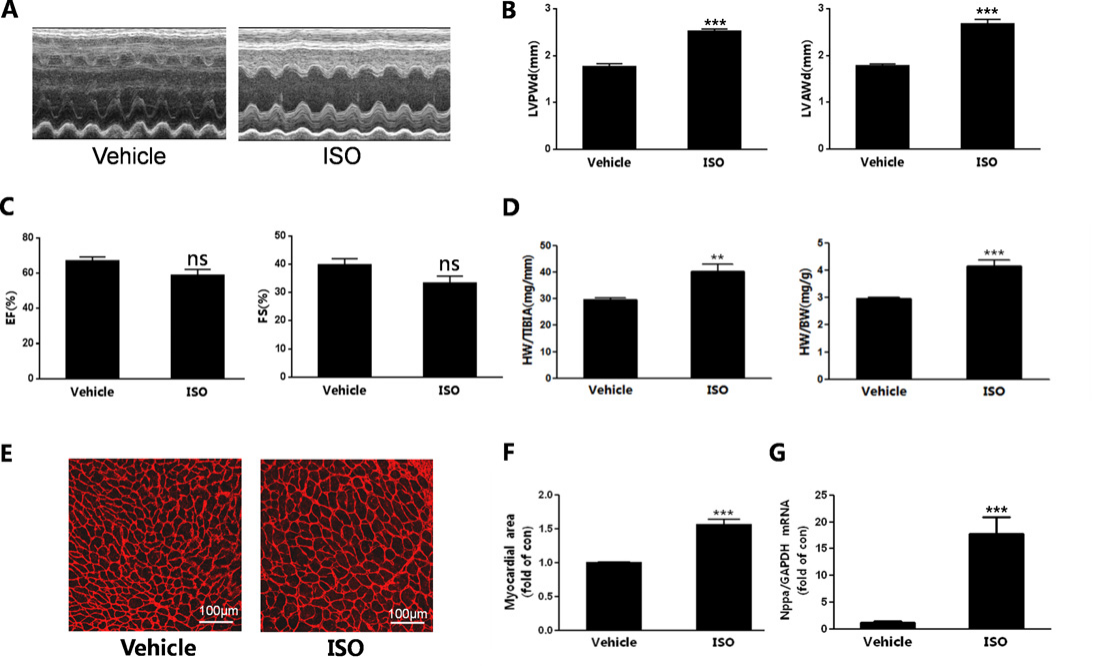
Cardiac hypertrophy induced by ISO stimulation. (A)Representative M-mode of LVs.(B)Echocardiography analyses of left ventricular wall thicknessat the end of diastole. LVAWd: LV anterior wall thickness at diastole, LVPWd: LV posterior wall thickness at diastole. (C)Measurement of ejection fraction(EF%) and fractional shortening(FS%). (D)Ratio of heart weight to tibia length(HW/TL)and heart weight to body weight(HW/BW). (E)Wheat germ agglutinin staining of transverse sections of hearts. Bars, 100μm. (F)Quantification of the size of cardiomyocytes by measuring transverse cell area. (G)The mRNA expression of ANP. n = 7, **P<0.01, ***P<0.001 ISO vs Vehicle.

Myocardial hypertrophy is the major structural change of cardiac remodeling. In the present study, we evaluated cardiac hypertrophy by using echocardiography. The results of echocardiography showed that LVPWd and LVAWd were increased by ISO(Fig 1B), as well as LVPWs and LVAWs (S1 Fig). We analyzed the ejection fraction (EF%) and fractional shortening(FS%).EF% and FS% were not decreased significantly by ISO, which indicated that cardiac models were remodeling (Fig 1C). In addition, heart weight to body weight ratio (HW/BW) and heart weight to tibia length (HW/TL) were used to evaluate cardiac hypertrophy. We found HW/BW and HW/TL were enhanced by ISO infusion(Fig 1D).

In addition, the size of myocytes and reactivation of fetal genes were detected to evaluate cardiac hypertrophy.ISO infusion increased the myocytes cross-sectional area(Fig 1E and 1F). The cell size was also assessed in isolated myocytes. ISO treatment for 24h and 48h increased myocytes size significantly (S2 Fig). The mRNA expression of ANP was significantly increased by ISO injection (Fig 1G).

These data suggested that myocardial hypertrophy was induced by subcutaneous administration of ISO(0.25mg/kg/d) up to seven days.

### Cardiac fibrosis induced by ISO stimulation

**Fig 2:**
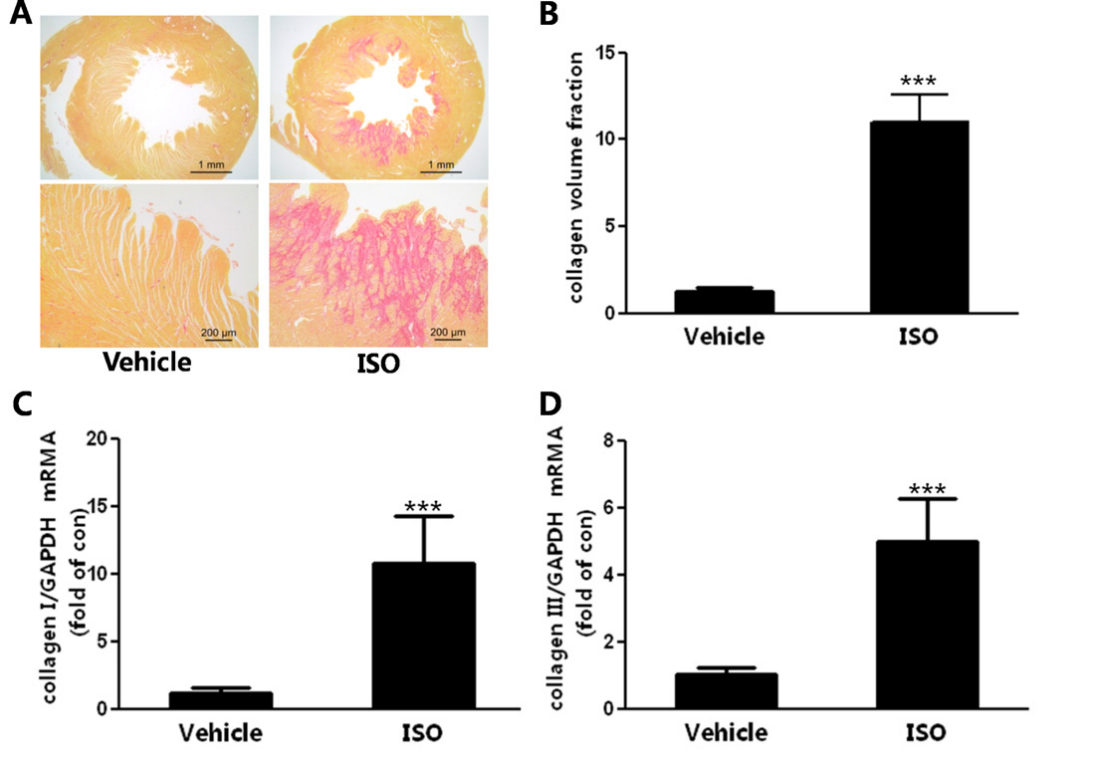
Cardiac fibrosis was induced ISO treatment. (A) Representative micrographs of picrosirius red-stained sections of the heart. Red parts represent collagen. (B) Quantification of cardiac interstitial collagen content from picrosirius red-stained sections with results expressed as collagen volume fraction. (C) Changes in the expression levels of mRNAs transcribed from collagen I. (D) The mRNA expression of collagen type III using real time PCR. n = 7, *** *P*<0.001 ISO vs Vehicle.

As previously mentioned, cardiac fibrosis is another structural change of cardiac remodeling. We quantitatively evaluated cardiac fibrosis with two methods, including robust morphological and biochemical assay. With picrosirius red staining, the collagen volume fraction of left ventricular was increased by ISO infusion (Fig 2A and 2B). The biochemical assay indicated that the mRNA expression of type I and III collagen were up-regulated by ISO injection(Fig 2C and 2D).

### The expression of β_1_-adrenoceptor was down-regulated in the remodeling heart

**Fig 3:**
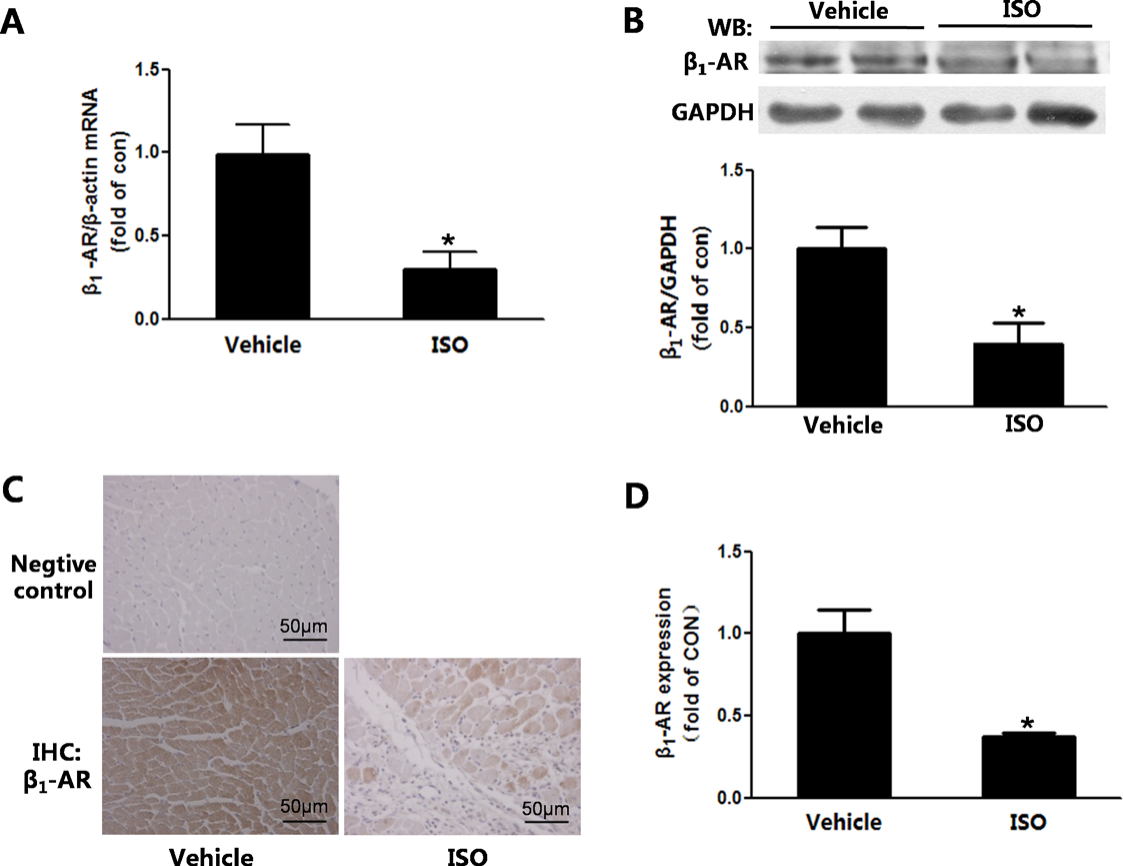
The β_1_-adrenoceptor expression was down-regulated in the remodeling heart. (A) The β_1_-AR mRNA expression was quantitated using real time PCR. (B)The expression ofβ_1_-AR was measured by western-blot.(C)Immunohistochemistry of β_1_-AR visualized in heart. (D)β_1_-AR expression was quantified from the immunohistochemistry sections of Fig 3C. Bar is 50μm for all fields. n = 7, * *P*<0.05 ISO vs Vehicle.

In cardiac remodeling induced by ISO, the mRNA expression of β_1_-AR was significantly reduced(Fig 3A). And the western-blot result showed that β_1_-AR expression decreased in the remodeling heart (Fig 3B). In addition, the immunohistochemistry result showed that the expression of β_1_-AR was decreased in the remodeling heart (Fig 3C and 3D).

### The expression of β_2_-adrenoceptor was down-regulated in the remodeling heart

**Fig 4:**
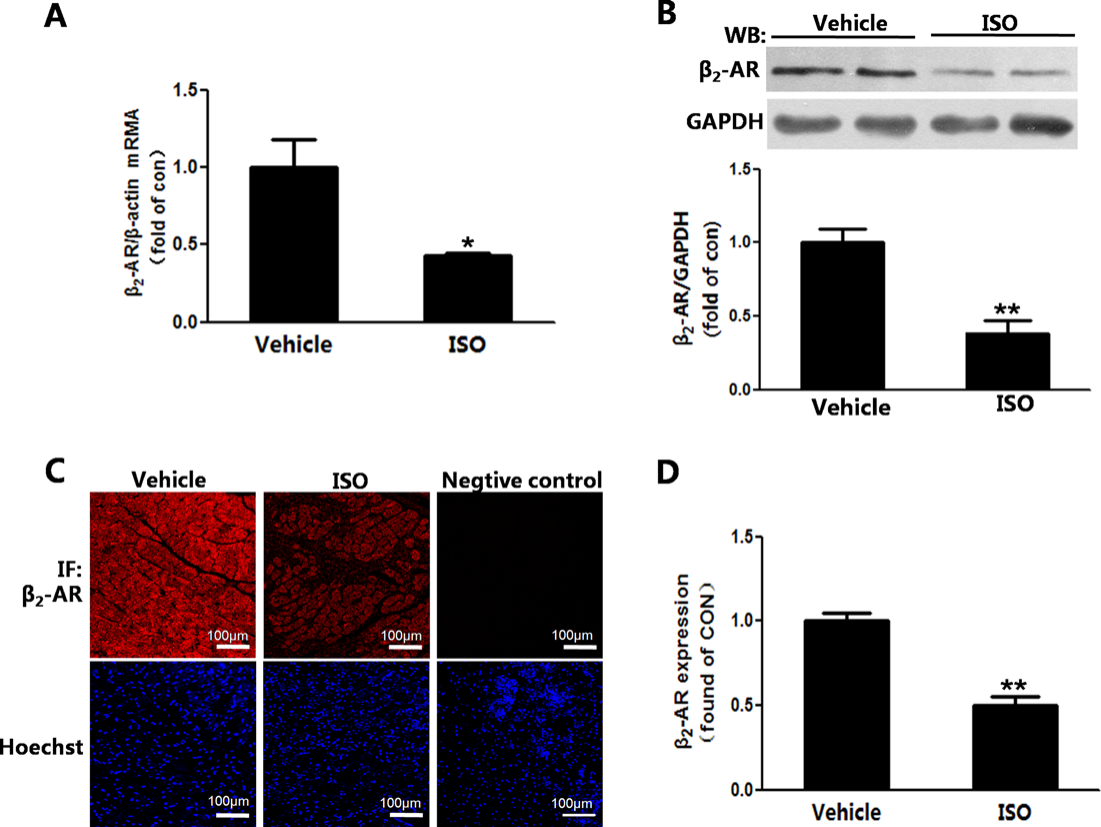
The expression of β_2_-adrenoceptor was down-regulated in the remodeling heart. (A)The mRNA level of β_2_-AR was measured by real time PCR. (B)The expression of β_2_-AR was detected by western-blot. (C)Immunofluorescence of β_2_ adrenoceptor visualized in heart. (D)β_2_-AR expression was quantified from the immunofluorescence sections of Fig 4C. Bar is 100μm for all fields. n = 7, *P<0.05, **P<0.01 ISO vs Vehicle.

Similar to β_1_-AR, the mRNA expression of β_2_-AR was significantly reduced in the remodeling heart(Fig 4A). And the western-blot result showed that β_2_-AR expression decreased in the remodeling heart(Fig 4B). The immunofluorescence results also showed that the expression of β_2_-AR was decreased in the remodeling heart (Fig 4C and 4D).

### The expression and activation of CREB were not affected in the remodeling heart

**Fig 5:**
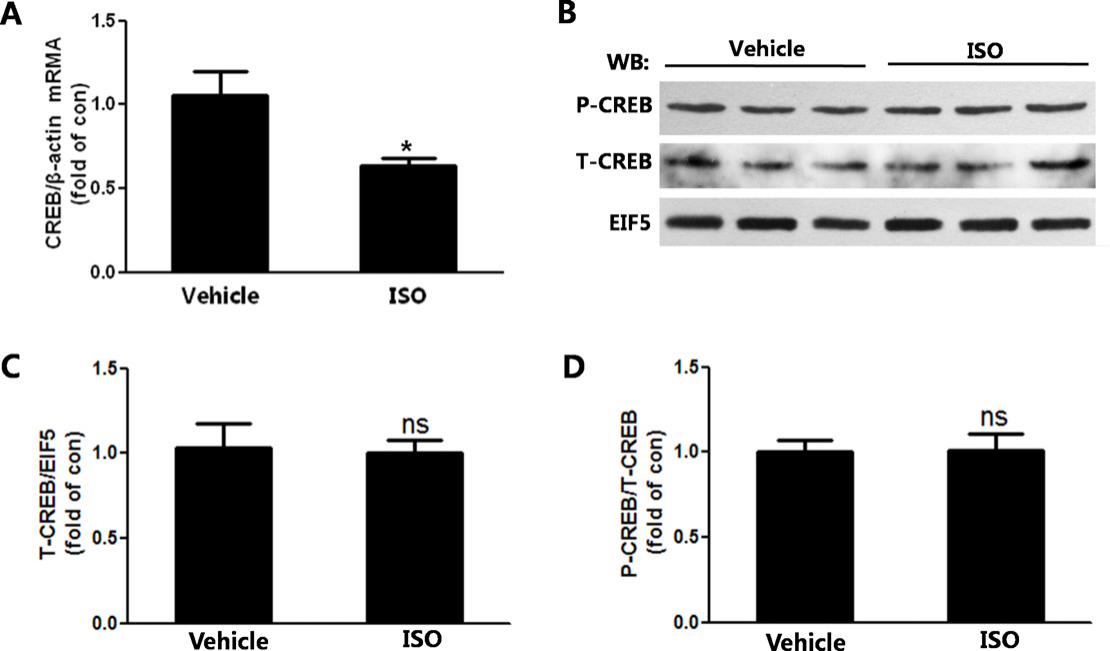
The expression and activation of CREB were not affected in the cardiac remodeling. (A)The mRNA expression of CREB measured by real-time PCR. (B)The protein expression of phospho-CREB, CREB measured by western-blot.(C) Quantification of CREB/EIF5 is shown. (D)Quantification of phospho-CREB/CREB is shown. n = 7, * *P*<0.05 ISO vs Vehicle.

To investigate the mechanism of the decrease of β-AR mRNA, CREB expression and activation was detected which regulated the transcription of β-AR. Although mRNA expression of CREB decreased (Fig 5A), the protein expression was not changed in the remodeling heart (Fig 5B and 5C). Further, the phospho-CREB level was not affected in the remodeling heart induced by ISO (Fig 5B and 5D).

### The expression of HuR was increased in the remodeling heart

**Fig 6:**
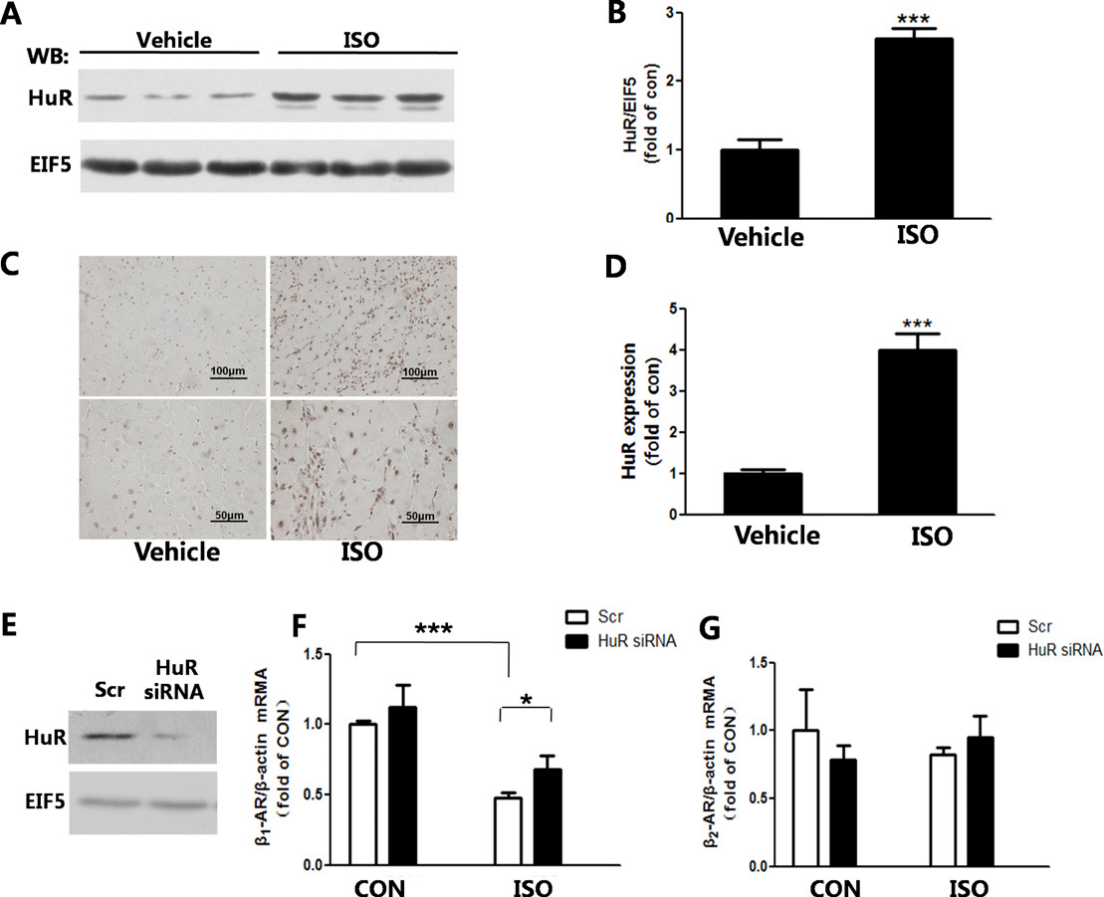
The expression of HuR was increased in the remodeling heart. (A)The expression of HuR measured by western-blot. (B)Quantification of HuR/EIF5 is shown.(C)Immunohistochemistry of HuR visualized in cell nuclei. (D)Quantification of HuR expression measured byimmunohistochemistryis shown. Five randomly selected fieldsfrom each section were collected to evaluate HuR expression using Image-Pro Plus. n = 7. ***P<0.001 ISO vs Vehicle. (E)Effect of HuR siRNA on HuR protein level. (F) The β_1_-AR mRNA expression was measured by q-PCR.With HuR knock down, NRCMs were induced by ISO(10^-5^M) stimulating for 48h. (G)β_2_-AR mRNA level was detected with deficiency of HuR induced by ISO in NRCMs. n = 4, * *P*<0.05,***P<0.001.

We detected HuR protein expression, which increased the degradation of β-AR mRNA at the post-transcriptional level. In our study, HuR protein expression was increased in the remodeling heart (Fig 6A and 6B). The immunohistochemistry result also showed that the expression of HuR in cell nuclei was raised in the remodeling heart (Fig 6C and 6D). In addition, we used siRNA to knock down HuR in NRCMs (Fig 6E). HuR deficiency significantly reversed the reduction of β_1_-AR mRNA induced by ISO stimulating for 48h (Fig 6F). In addition, deletion of HuR partially reversed the decrease of β_2_-AR mRNA induced by ISO (Fig 6G).

## Discussion

Cardiac remodeling appears in many pathologic conditions including myocardial infarction, hypertension, hypertrophic cardiomyopathy, dilated cardiomyopathy and diabetic cardiomyopathy[22]. The excessive activation of sympathetic nervous system contributes to cardiac remodeling and the progression of above pathological conditions. Chronic sympathetic activation in myocardium promoted the increase of left ventricular mass and cardiac noradrenaline spillover in patients with essential hypertension[23]. Clinical research also exhibited the increase in plasma norepinephrine with acute myocardial infarction[24]. The sympathetic transmitter, NE, binds specifically to ARs, which consist of 9 subtypes, 3 α_1_-ARs (α_1A_, α_1B_ and α_1D_), 3 α_2_-ARs (α_2A_, α_2B_and α_2C_), and 3 β-ARs (β_1_, β_2_and β_3_)[25]. Activation of β_1_- and β_2_-AR, the major subtypes in heart, is a main mechanism in the progression of cardiac remodeling[26]. Thus, we used the infusion of isoproterenol, a non-selective β-AR agonist, to build the model of cardiac remodeling including ventricular hypertrophy and interstitial fibrosis.

Besides effectors, gene expressions of β-ARs were regulated by stress stimulation[27]. In the present study, our results showed that both β_1_-AR and β_2_-AR mRNA expression was down-regulated in cardiac remodeling model. Consistent with our result, β_1_-AR and β_2_-AR mRNA decreased by 30% and 42% respectively with isoproterenol treatment in H9C2 cell[28]. In patients, β-AR density decreased in the remote non-infarcted region after prior myocardial infarction with left ventricular remodeling[29].Cardiac remodeling is a compensation process, in which the expression of β-ARs is down-regulated to prevent the receptor from over-activation in the remodeling heart. However, another study showed β_2_-AR was constant in the failing heart[30]. The possible reason is that heart failing is already a decompensation stage and accompanied bysome complicated pathophysiological processes. In this stage, the receptor was not necessary to desensitize through down-regulating its expression.

CREB, binding to the cAMP response element[31], mediated the gene expression induced by activated ARs[32].CREB mediated the JHDM2a expression in porcine tissues and cells with stimulation of Clenbuterol, β_2_-AR agonist[33]. In response to the β-AR activation, CREB also functioned as transcription factor and regulated the expression of pro-inflammatory cytokines such as IL-6[34].Further, the regulation of AR gene expression is mediated by the second messenger cAMP in a feedback form, which indicates that CREB can regulate the AR expression with agonist stimulating. ISO treatment decreased β_1_- and β_2_-AR mRNA expression and CREB was involved in this phenomenon in rat lung[35].The phosphorylation of CREB was also found to be increased by ISO stimulating for 5 minutes in rat cardiac fibroblasts[36]. However, our study showed that both phosphorylated and total CREB were not changed in the rat heart with ISO administration for 7 days. The possible reason for this was associated with the duration of catecholamine stimulation. Previous studies demonstrated that the short-term stimulation with catecholamines activated cAMP and increased CREB mRNA and phosphorylation of CREB[36, 37]. However, the prolonged catecholamine stimulation led to the desensitization of cardiac β-ARs, which limited cAMP generation and reduced the expression and phosphorylation of CREB[38, 39].

The post-transcriptional regulation was another major way to control the expression of β-ARs. HuR is an important protein to regulate gene expression at the post-transcriptional level, which could regulate the mRNA stability of β-AR. HuR, consisting of four members, HuA(HuR),HuB, HuC and HuD, binds to AUUUA pentamers or derivative sequences[40].HuR recognizes the untranslated regions (UTRs) of transcripts to regulate mRNA stability[41]. Contrast to the previous study which suggested HuR promoted mRNA stability[42, 43], HuR negatively regulated mRNA stability in the present study. HuR expression is responsible for the decrease of cyclin D1 mRNA stability by suberoylanilide hydroxamic acid stimulating[44].HuR also played an important role in the agonist-mediated downregulation of β_1_-AR[45],and was critical for translational suppression of β_2_-AR[46]. In our study, HuR expression was increased in the remodeling heart, and HuR deficiency reversed the reduction of β_1_-AR mRNA induced by ISO treatment. Meanwhile, β_2_-AR expression was regulated partially through HuR. It is possible that β_2_-AR expression was regulated by other mechanisms, like receptor degradation [47].

**Fig 7:**
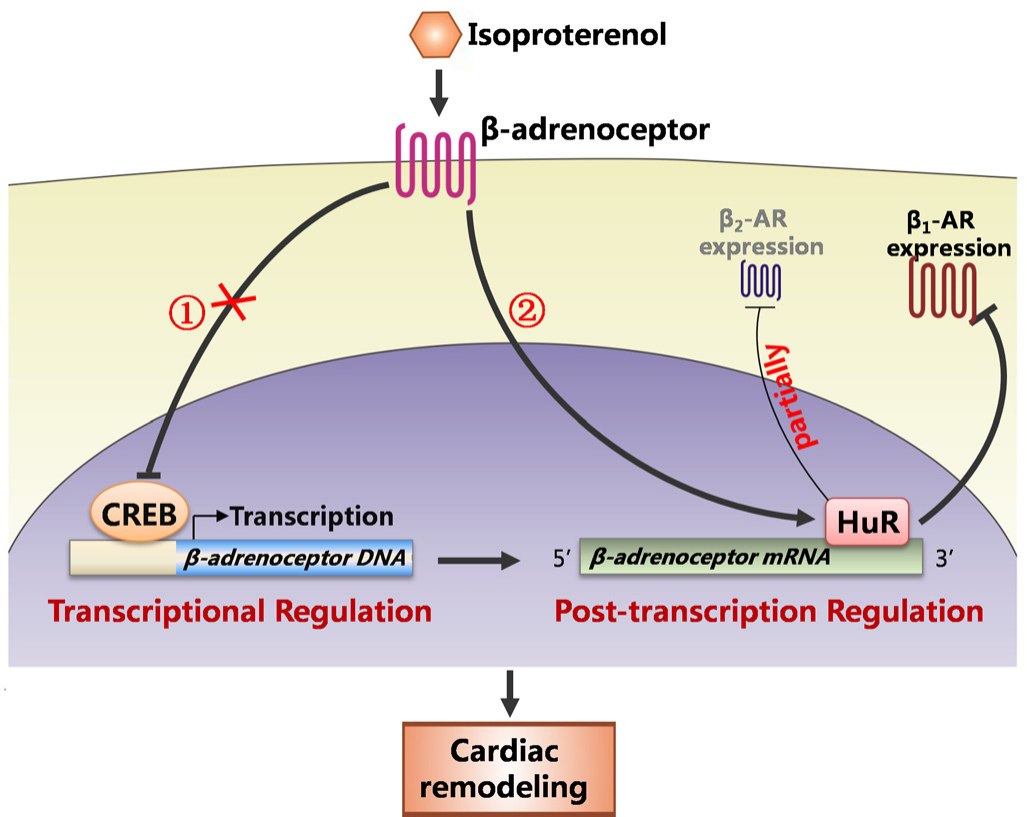
Graphic summary for how the β-ARs were down-regulated in ISO-induced cardiac remodeling. Sustained β-AR activation by isoproterenol enhanced HuR expression rather than inhibited CREB expression and activation, which decreased the β_1_-AR expression and partially down-regulated the β_2_-AR expression in the cardiac remodeling model.

In the present study, cardiac remodeling was successfully induced by sustained administration of ISO. In this model, the expression of β_1_- and β_2_- AR is decreased, which is regulated by HuR at post-transcriptional level rather than by CREB at transcriptional level (Fig 7).

## Supporting Information

S1 Fig Echocardiography analyses of left ventricular wall thickness at the end of systole. (A) LVPWs: LV posterior wall thickness at systole. (B) LVAWs: LV anterior wall thickness at systole.
(TIF)

S2 Fig The myocytes size was increased by ISO in NRCMs. (A)The myocytes size was evaluated. The myocytes was stimulated with ISO for 12h, 24h and 48h. (B)The myocytes size was quantified for S2A Fig. n = 4, * P<0.05, *** P<0.001 vs CON.
(TIF)

## Abbreviations

AR: adrenergic receptor
CREB: cAMP-response element binding protein
CVF: collagen volume fraction
HW/BW: heart weight to body weight ratio
HW/TL: heart weight to tibia length
HuR: Hu antigen R
ISO: Isoproterenol
LVAWd: LV anterior wall thickness at diastole
LVAWs: LV anterior wall thickness at systole
LVPWd: LV posterior wall thickness at diastole
LVPWs: LV posterior wall thickness at systole
WGA: wheat germ agglutinin
