# Supplementary figures and images for: Downregulation of β-Adrenoceptors in Isoproterenol-Induced Cardiac Remodeling through HuR
Source: PLoS One. 2016 Apr 1;11(4):e0152005. doi: 10.1371/journal.pone.0152005 (PMC4818026; doi:10.1371/journal.pone.0152005)

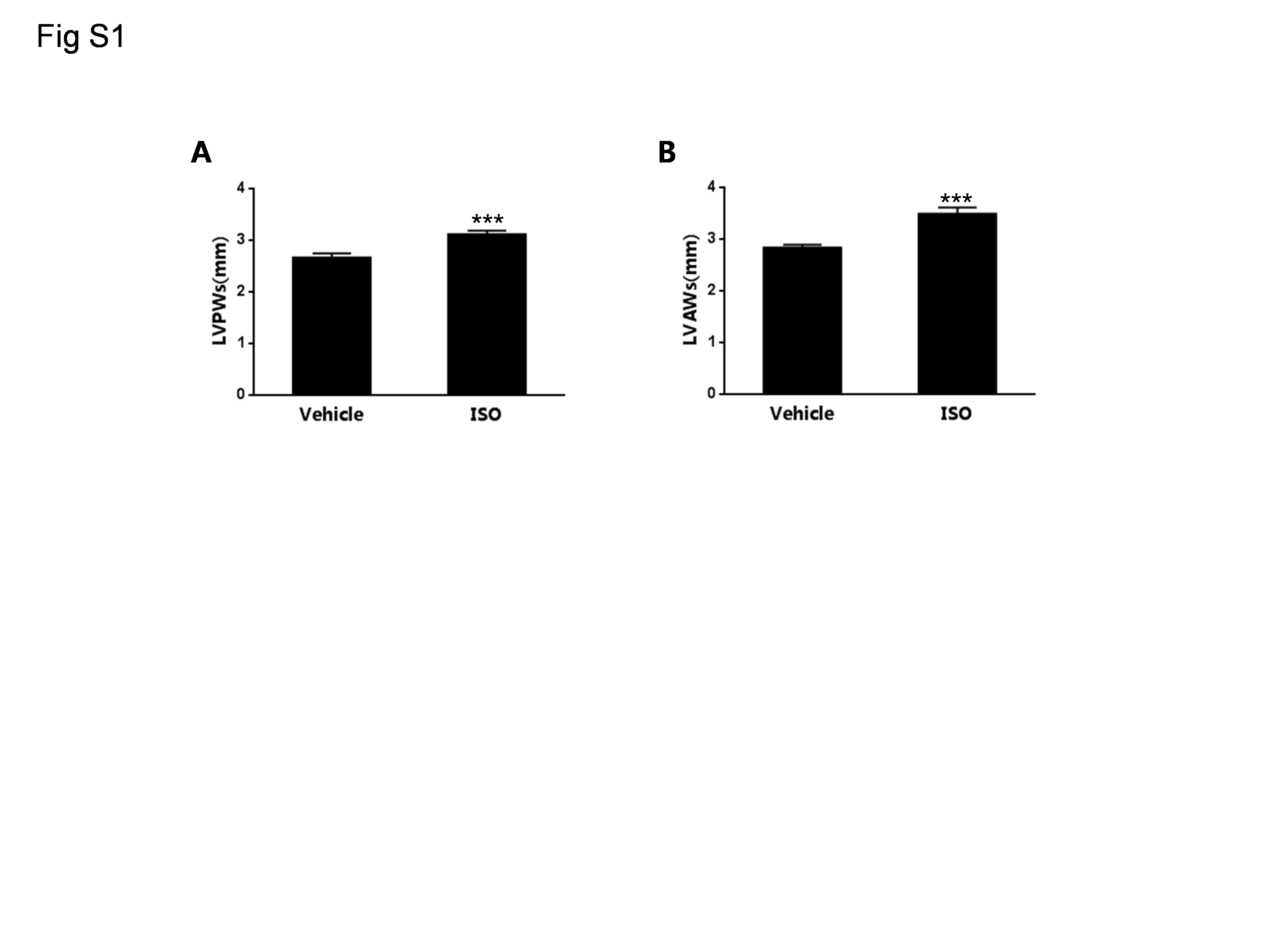

Supplement: S1 Fig — (A) LVPWs: LV posterior wall thickness at systole. (B) LVAWs: LV anterior wall thickness at systole. (TIF) [file pone.0152005.s001.tif]

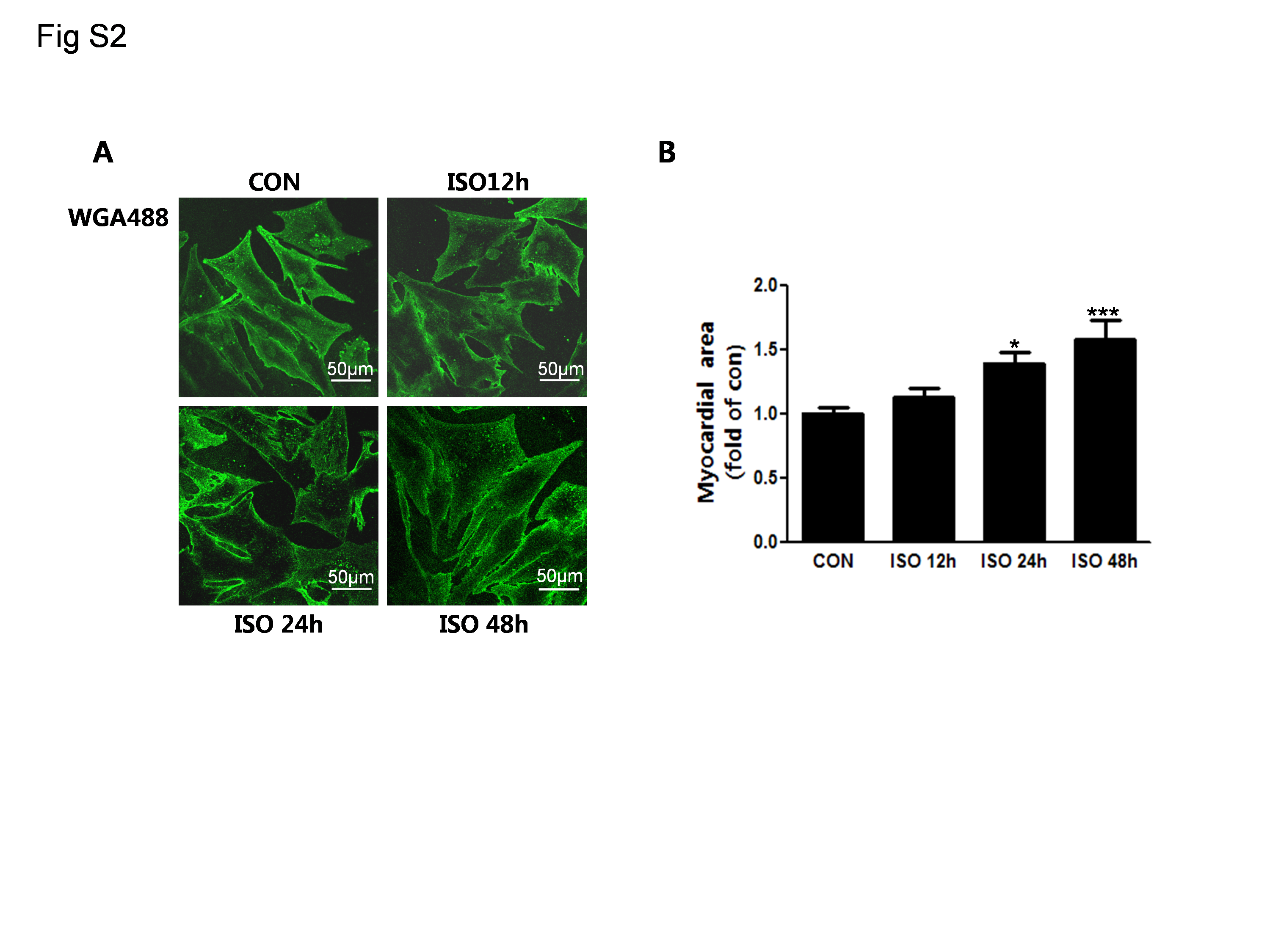

Supplement: S2 Fig — (A)The myocytes size was evaluated. The myocytes was stimulated with ISO for 12h, 24h and 48h. (B)The myocytes size was quantified for S2A Fig. n = 4, * P<0.05, *** P<0.001 vs CON. (TIF) [file pone.0152005.s002.tif]
